# Applying Theory to Explain the Influence of Factors External to an Organization on the Implementation of an Evidence-Based Intervention

**DOI:** 10.3389/frhs.2022.889786

**Published:** 2022-05-26

**Authors:** Jennifer Leeman, Mary Wangen, Michelle Kegler, Matthew Lee, Meghan C. O'Leary, Linda K. Ko, María E. Fernández, Sarah A. Birken

**Affiliations:** ^1^School of Nursing, University of North Carolina at Chapel Hill, Chapel Hill, NC, United States; ^2^Center for Health Promotion / Disease Prevention, University of North Carolina at Chapel Hill, Chapel Hill, NC, United States; ^3^Department of Behavioral, Social and Health Education Sciences, Rollins School of Public Health of Emory University, Atlanta, GA, United States; ^4^Department of Population Health, New York University Grossman School of Medicine, New York, NY, United States; ^5^Department of Health Policy and Management, University of North Carolina at Chapel Hill, Chapel Hill, NC, United States; ^6^Health Systems and Population Health, School of Public Health, University of Washington, Seattle, WA, United States; ^7^Health Promotion and Behavioral Sciences, University of Texas Health Science Center at Houston, Houston, TX, United States; ^8^Department of Implementation Science, Wake Forest School of Medicine, Wake Forest University, Winston-Salem, NC, United States

**Keywords:** organizational theory, implementation determinants, evidence-based interventions, cancer prevention and control, implementation strategies

## Abstract

Despite its widely acknowledged influence on implementation, limited research has been done on how the external environment (i.e., outer setting) determines when organizations adopt and implement new interventions. Determinant frameworks identify several outer setting-level factors such as funding streams, inter-organizational relationships, and peer pressure. However, these frameworks do not explain how or why outer-setting factors influence implementation. To advance research in this area, we argue for the importance of deriving theory-based propositions from organization theory to explain how outer setting factors influence organizations. Drawing on the work of the Organization Theory in Implementation Science (OTIS) project, we identified 20 propositions from five classic organization theories—Complexity Theory, Contingency Theory, Institutional Theory, Resource Dependence Theory, and Transaction Cost Economics. We then applied those propositions to hypothesize relationships among outer setting factors, implementation strategies, and implementation outcomes in five case studies of evidenced-based tobacco control interventions. The five case studies address the implementation of smoke-free policies, community health worker-led tobacco education and cessation programs, 5 A's (Ask, Advise, Assess, Assist, and Arrange), point-of-sale tobacco marketing policy interventions, and quitlines. The case studies illustrate how propositions may be used to guide the selection and testing of implementation strategies. Organization theories provide a menu of propositions that offer guidance for selecting and optimizing high-leverage implementation strategies that target factors at the level of outer setting. Furthermore, these propositions suggest testable hypotheses regarding the mechanisms underlying the influence of outer-setting factors on how and why organizations adopt and implement interventions.

## Introduction

Implementation scientists continue to study new and better ways to accelerate the implementation of evidence-based interventions (EBIs) into practice by designing strategies to target the multilevel factors (i.e., determinants) that influence implementation (1). Despite these efforts, a recent study of five cancer control EBIs found they took an average of 15 years to achieve wide scale implementation (2). The slow rate of EBI implementation may relate in part to the relatively limited attention implementation scientists have given to how environmental factors influence organizations. To date, implementation scientists have focused on determinants at the level of the individuals who adopt and implement EBIs and the inner setting of the organizations where they work, with less attention to determinants at the level of the external environment or outer setting (3, 4). In this paper, we argue for the value of organization theory as a means of closing this gap.

Within organization theory, organizations are conceptualized as goal-directed, social entities that are influenced by their environments (5). Organization theories include environmental variables and propositions that explain how those variables influence the organization as a unit. As such, these propositions offer a rich resource for implementation scientists to use as a guide to selecting implementation strategies and hypothesizing the causal pathways or mechanisms through which those strategies affect proximal outcomes (e.g., changes to the organization) and more distal outcomes (adoption and implementation of EBIs), as well as barriers and facilitators that may moderate the strategies' impact on those outcomes (6).

In this paper, we build on the work of the organization Theory in Implementation Science (OTIS) project (https://cpcrn.org/projects) to describe how propositions from five classic organization theories might be applied to select implementation strategies and hypothesize relationships among outer-setting determinants, implementation strategies, and implementation outcomes. The focus on classic theories addresses Kislov et al.'s recommendation that implementation scientists draw on grand or classic theories as one starting point for theorizing mechanisms underlying implementation (7).

### Organization Theory for Implementation Science (OTIS)

The OTIS project aims to identify organization theories relevant to implementation and extract and summarize their constructs and propositions. OTIS project methods are described elsewhere (8). Briefly, we surveyed scholars with expertise at the intersection of implementation and organization science and through that survey identified nine organization theories relevant to implementation science. Two members of the team then abstracted information about the theories from seminal texts. Two members of the team then summarized information about the theory into a structured template that includes the theory's central constructs and propositions together with guidance on relevant implementation strategies. Summaries of each of the nine theories are available on the Cancer Prevention and Control Research Network's (CPCRN) website (https://cpcrn.org/resources). The purpose of this paper is to illustrate how propositions from these organization theories might be used to hypothesize relationships among outer-setting factors, implementation strategies, and implementation outcomes. Building on co-authors' expertise, we selected a subset of five organization theories and illustrated their use in case studies of the implementation of tobacco control EBIs.

## Five Organization Theories and Their Propositions

Table 1 presents central propositions from five classic organization theories: Complexity Theory, Contingency Theory, Institutional Theory, Resource Dependence Theory, and Transaction Cost Economics. Below we describe each theory and provide a case study to illustrate the theory's application. In proposing relevant implementation strategies, we named strategies using terminology developed by the Expert Recommendations for Implementing Change (ERIC) project (10).

**Table 1:** A partial list of propositions for each of five organizations theories.

**Theory**	**Propositions**
Complexity theory	• Interdependencies contribute to sense making. • Interdependencies that are “trusting, attentive to new ideas, and mindful of differences between ideas” are most likely to result in effective sense making [Lanham et al. (9) as cited in Lanham et al. (9)] • Interdependencies and sense making contribute to self-organization. • Change that is guided by minimum specifications allows individuals to self-organize most effectively. • Feedback loops may amplify some effects and reduce others, and therefore small changes may lead to large-scale differences in outcomes (i.e., “the butterfly effect”) and vice versa.
Contingency theory	• Optimal work structure is contingent on the degree of uncertainty in both the task and in the task environment. • When there is higher uncertainty in a setting, unprogrammed (more flexible) coordination structures will be more effective. • When there is lower uncertainty in a setting, programmed (less flexible) coordination structures will be more effective. • Higher levels of interdependence (both within and between departments) will require greater investment in coordination. • The greater the differentiation between departments, the more difficult it will be to coordinate.
Institutional theory	• The degree of isomorphism in an organizational field is positively related to the degree of (1) coercive, (2) mimetic, and (3) normative pressures in that field. • Coercive pressures are greater to the extent that: - Organizations in a field transact with agencies of the state (or depend on public financing). - Organizations in a field are dependent upon a single (or several similar) source of support for vital resources. • Mimetic (i.e., peer) pressures are greater when an organizational field has high levels of uncertainty (e.g., evidence for what is effective is limited, technologies are poorly understood, goals are ambiguous, etc.). • Normative processes are greater in organizations with higher levels of professionalization.
Resource dependence theory	• To acquire power, organizations exchange their autonomy for resources from other organizations within their field. • Multiple environmental factors influence an organization's willingness to exchange autonomy for power, including competition, interdependence, and munificence.
Transaction cost economics	• Organizations incur costs when they transact with other organizations for goods and services (transaction costs). • Organizations strive for greater efficiency by implementing governance structures that will minimize transaction costs. • These governance structures range from (a) buying the good or service with no contract, (b) contracting with another organization to provide the good or service, and (c) integrating production within the organization (i.e., producing the good or service themselves). The following characteristics of a transaction determine the optimal type of governance structure: ° asset specificity (i.e., investment of personnel, materials, and other resources required to establish the transaction) ° transaction frequency ° transaction complexity ° uncertainty about future transactions • Integrating production will be more efficient than transacting with other organizations to produce a good or service to the extent ath asset specificity is high and transactions are infrequent, uncertain, and complex.

### Complexity Theory

Complexity Theory explains how change occurs within complex systems that are comprised of diverse yet interconnected parts that affect and influence each other in dynamic ways over time (11). As organizations interact with others in their network and develop relationships (i.e., interdependencies), they learn from each other, adapt behaviors, engage in sense-making (in which they assign meaning to their collective experiences), and develop patterns of organization (i.e., self-organization) unique to their system. Outputs of a system process may become inputs within a chain of cause-and-effect that forms a loop (i.e., feedback loops). Feedback loops influence the magnitude of effects and, given the dynamic interactions occurring within the system, can create paradoxical effects; small changes may have large effects on outcomes and large changes may have small effects.

Complexity Theory offers possible mechanisms for developing and fostering interactions and social processes to optimize EBI implementation within a system. Hypothesized mechanisms for facilitating effective sense-making include developing interconnections among those with diverse perspectives to promote trust, innovation, and respect for differences and thereby build support for an EBI (9). Minimum specifications, or flexible rules that allow for innovation, are posited to increase the effectiveness of self-organization and thus improve EBI adaptation and integration to fit the parameters of a complex system (11). Prior studies have called for applying Complexity Theory to EBI scale-up and spread across systems (9) and conducting complexity-informed implementation science (12).

#### Illustration: Complexity Theory Applied to Implementation of Smoke-Free Housing Policies

Mills and colleagues' causal loop diagram of individual, environmental, and root causes influencing disparities in smoking rates (13) visualizes the complexity surrounding tobacco control efforts. Their diagram illuminates pathways to explain how implementing smoke-free policies in multi-unit housing could decrease smoking, and possible unintended effects that could sustain or exacerbate smoking disparities. For example, enforcement of smoke-free policies may lead to evictions and threats of eviction, which may contribute to housing instability, stress, and anxiety, thereby increasing smoking rates (14, 15). Complexity Theory suggests the value of implementation strategies that foster interactions among the multiple other organizations that support the residents of multi-unit housing (e.g., housing advocates, public health departments, housing authorities, and eviction courts). These strategies might include building a coalition to capture and share local knowledge, engage in local consensus discussions, and facilitate sense-making to develop and plan for implementation of smoke-free policies with the goal of maximizing public health outcomes (e.g., decreased smoking, decreased secondhand smoke exposure, stable housing). Such inter-organizational collaborations can also formalize feedback loops for improved decision-making by the system and flexibility in policy implementation (e.g., outdoor designated smoking areas to improve compliance among smokers; provision of nicotine tobacco replacement therapy and Quitline referrals as part of the violation response process).

### Contingency Theory

Contingency Theory posits that there is no best way for organizations to operate but rather, the most effective or optimal way for an organization to structure and coordinate tasks is contingent on characteristics, particularly the level of uncertainty, of both the task and the task environment (16–18). Uncertainty in the task refers to gaps between the information needed vs. information available to perform the task. Uncertainty in the task environment (inner and outer setting) refers to the degree that factors in the environment are predictable (e.g., to what extent and how quickly are changes happening in the evidence-base, resource availability, community needs, or guidelines and policies). Depending on the degree of uncertainty, different strategies will be best suited to coordinate a task. Programmed/inflexible approaches to coordination will be optimal when uncertainty is low and less programmed/flexible approaches will be optimal when uncertainty is high (19). Thus, the effectiveness of an organization's actions are contingent upon the organization's dynamic internal and external contexts, which also continuously shape the organization's structure and development.

While still underutilized in implementation science, researchers have begun to identify potential applications of Contingency Theory. For example, some authors have linked Contingency Theory to advancements in adaptation research. Since organizational structure is a critical determinant of implementation success, they suggest regularly revisiting and adapting implementation strategies to fit how organizations are continuously altering their structure in response to dynamic factors in the inner and outer setting (20). In another example, researchers applied Contingency Theory to inform a study of strategies that foster cross-systems collaborations between child welfare and substance use treatment agencies (21).

#### Illustration: Contingency Theory Applied to Implementation of Community Health Worker led Tobacco Education and Cessation Programs

We use an example of a coalition of community-based health care and social services organizations that aimed to address gaps in local tobacco control implementation and growing inequities in smoking prevalence and smoking-related cancer among immigrant communities. To improve the reach and effectiveness of tobacco education and cessation programs, the coalition shared local knowledge and built collective capacity to implement intervention strategies, including leveraging community health workers (CHWs). Coalition members have varying capacities to adopt, implement, and sustain a tobacco specialist CHW program. Contingency Theory can be used to identify and monitor risks, vulnerabilities, and capacity to inform planning and implementation among coalition members. For example, assessing characteristics of the task and task environment of member organizations (e.g., organization size and budget, staff turnover, prior experience delivering CHW-led programs) may offer coalition leaders insight into potential sources of uncertainty. Some organizations may face higher uncertainty related to the task (e.g., hiring and training CHWs) while others may face higher uncertainty related to the task environment (e.g., stability of funding, shifting community priorities). Concretely assessing and monitoring these uncertainties will inform how the coalition implements the CHWs intervention to align with the capacity of each organization. In cases of low uncertainty, the coalition may establish standardized protocols for the roles of CHWs and their supervisors. Alternatively, in cases of high uncertainty, the focus may shift to promoting adaptability, quality monitoring, and small tests of change with the goal of supporting organizations and CHWs to develop the best local approach to implementation. By acknowledging areas where either more programmed or unprogrammed coordination will be more effective, member organizations will be better positioned to benefit from participation in the coalition and have opportunities to select tailored strategies that best meet their organizational context and ideally lead to stronger and more sustainable collaboration.

### Institutional Theory

Institutional Theory hypothesizes that organizations within a field (e.g., regional healthcare market) become increasingly similar (i.e., ‘isomorphic’) as a result of mimetic, normative, and coercive pressures (22). Mimetic pressures are evident when organizations copy the approaches of others within their field; the greater the uncertainty about which approaches are best, the greater the mimetic pressure. Normative pressure comes from institutions that legitimize a field (e.g., professional societies) and is greatest in highly professionalized fields such as healthcare (20, 23, 24). Jensen et al. and Sherer used Institutional Theory as a lens for understanding how the ‘rationalized myth’ of electronic health records promoted their adoption and implementation (25, 26). Burnett et al. used Institutional Theory to explain hospitals' responses to often conflicting pressures to improve quality and constrain spending (27). Birken et al. used Institutional Theory to explain how child welfare systems responded to demands from policymakers for evidence-based solutions to child abuse and neglect by adopting SafeCare, a widely vetted intervention (20).

#### Illustration: Institutional Theory Applied to Implementation of the 5 A's in a Network of Community Health Clinics

The “5 A's” specifies five steps (Ask, Advise, Assess, Assist, and Arrange) to identify tobacco users and either provide or refer them to interventions (28–30). A network of community health clinics seeking to implement the 5 A's could leverage institutional pressures by partnering with accrediting bodies and payers to require clinics to use 5 A's to meet quality standards or funding requirements. Mimetic pressure may be invoked by forming a learning collaborative to increase awareness of 5 A's use among peer organizations. Partnering with professional organizations or highlighting their endorsement of the 5 A's may leverage normative pressures among healthcare providers.

### Resource Dependence Theory

Resource Dependency Theory (RDT) describes how procurement of external resources by an organization affects the strategic and tactical management of the organization. Most notably, RDT predicts the conditions under which organizations will compromise autonomy to gain power. An organization's power may include not only its financial standing but also its prestige and reputation (31). RDT identifies multipe environmental factors that influence when an organization will trade autonomy to gain power including competition within the external environment, interdependence with other organizations, and munificence (i.e., richness of resources), among others (32). RDT has been used to understand the relationship between an organization and its external environment including strategies, structure, and/or performance in both healthcare and non-healthcare settings (33–36). For example, Fareed & Mick used RDT to hypothesize that more interdependent hospitals in munificent environments would be more likely to engage in patient safety innovations than hospitals with fewer dependencies and less munificent environments (31).

#### Illustration: Resource Dependence Theory Applied to Implementation of Point-of-Sale Tobacco Marketing Policy EBIs

RDT aids in understanding the dynamic, interdependent relationships among organizations that compete for resources, such as state health departments and other community organizations interested in changing point-of-sale tobacco marketing policies. Community organizations often must apply for government and foundation funding to finance the implementation of programs. External funding enables organizations to access training, technical assistance, and software needed to collect, manage, visualize, and analyze data on tobacco marketing practices, and the locations of tobacco retailers (e.g., near schools) (37, 38). Tobacco retailer data can be shared with local policy makers, giving community organizations increased power to inspire change to promote public health. At the same time, relying on external funding may limit organizations' autonomy as they comply with funding requirements. They may also need to increase their interdependence with other organizations to garner the expertise and resources needed to collect local data and promote policy change. In exchange for this expertise and resources, organizations may need to adjust the direction of their work, make changes to their timeline, or institute other changes to meet the needs of other organizations upon which they depend for resources, thus further decreasing their autonomy. Community organizations may limit the loss of autonomy and protect their power through the use of implementation strategies such as resource sharing agreements, formal commitments, and shared timelines.

### Transaction Cost Economics

Organizations incur costs as a result of planning, implementing, and enforcing transactions with other organizations to exchange goods and services. Transaction Cost Economics (TCE) posits that organizations will transact with other organizations to produce a good or service (“buy” from another organization instead of “make” in-house) when the transactions required to do so are less than the cost of producing the good or service in-house (39, 40). Several authors have argued for the relevance of TCE to healthcare generally and to implementation science more specifically. For example, authors have argued that TCE could be applied to explain why some Accountable Care Organizations vertically integrate a service (e.g., adding rehabilitation services within the Accountable Care Organization) while others opt to purchase the service from an external organization (41). In another case, authors argued that TCE might explain when a health insurer would hire their own case managers to provide Diabetes Self-Management Education (DSME) as opposed to reimbursing community practices to provide DSME services (42).

#### Illustration: Transaction Cost Economics Applied to Quitline Implementation

Tobacco quitlines are an EBI that has been shown to increase tobacco cessation rates, particularly when quitline counselors proactively call participants to provide multiple counseling sessions (43). To increase referrals to the quitline, a state department of health and human services (DHHS) might explore whether it would be more efficient to work within their own network of health departments (internal integration) or with the state's federally qualified health centers (FQHCs) (external transaction). This decision could be viewed through the lens of TCE. For either health departments or FQHCs, DHHS would need to invest personnel time and other resources in training, technical assistance, and performance monitoring. The value of this investment would depend on the frequency of potential referrals, complexity of transactions, and uncertainty of continuing the transactions over time. FQHCs may have the potential to generate more frequent referrals but have higher levels of complexity and uncertainty. The most efficient choice would depend on how many more referrals FQHCs would generate compared to health departments, and whether the additional referrals merited the higher levels of complexity and uncertainty. TCE also could be used to identify factors that might moderate the impact of efforts to increase referral rates. This might include increased uncertainty about whether transactions will continue due to potential reductions in funding. TCE might inform implementation strategies related to formal commitments between transacting organizations and efforts to increase demand for a service (e.g., by marketing the quitline), which would increase the frequency of transactions.

## Discussion

As we have illustrated in this paper, organization theories offer propositions that may be used to better understand how, when, and why outer setting-level determinants influence organizations' adoption and implementation of EBIs. These propositions can also guide the selection of implementation strategies and generate testable hypotheses about the mechanisms through which those strategies affect implementation outcomes. As summarized in Table 2, organization theories may inform the selection and testing of a wide range of implementation strategies.

**Table 2:** Propositions from organizational theories aligned with implementations strategies.

**Theory**	**Central propositions**	**Relevant implementation strategies [Powell et al. (1)]**
Complexity theory	Systems are complex. Facilitating interdependencies and sense making contributes to self-organization within complex systems.	• Build a coalition • Capture and share local knowledge • Conduct cyclical small tests of change • Conduct local consensus discussions • Create new clinical teams • Facilitate relay of clinical data to providers • Model and simulate change • Develop and organize quality monitoring systems • Organize clinician implementation team meetings • Promote adaptability • Promote network weaving • Purposefully re-examine the implementation
Contingency theory	When task and/or environmental uncertainty are high, unprogrammed coordination will be most effective.	
	When task and/or environmental uncertainty are low, programmed coordination will be most effective.	• Mandate change • Provide clinical supervision
Institutional theory	The degree of isomorphism in an organizational field is positively related the degree of (1) coercive, (2) mimetic, and (3) normative pressures in that field.	• Alter incentive/allowance structure • Change accreditation or membership requirements • Create a learning collaborative • Create or change credentialing and/or licensure standards • Use capitated payments • Visit other sites • Place innovation on fee for service lists/formularies
Resource dependence theory	To acquire power, organizations exchange their autonomy for resources from other organizations within their field.	• Develop resource sharing agreements • Obtain formal commitments • Develop academic partnerships • Fund and contract for the clinical innovation • Increase demand
Transaction cost economics	Integrating production (making the product or service) will be more efficient than transacting with other organizations to produce it to the extent that asset specificity is high and transactions are infrequent, uncertain, and complex.	

Our presentation of the five organization theories is not intended to be comprehensive but rather to illustrate the potential of organization theory to advance implementation research related to the influence of outer setting-level determining when and how organizations adopt and implement EBIs. We provide only a broad level overview of the five theories, each of which has a long history that includes multiple permutations with varying constructs and propositions. We also recognize that implementation scientists face barriers to studying the impact of outer setting determinants on implementation that extend beyond gaps in knowledge of organization theory. These barriers include, but are not limited to, difficulties manipulating outer setting variables, controlling exposure to outer setting variables across study arms, and garnering the sample sizes needed to test hypotheses related to the influence of outer setting variables on organizaitons.

This paper represents one piece of our broader effort to make classic theories more accessible to implementation scientists, propelling the field toward improved success in translating evidence into practice. Without theory, the mechanisms that drive implementation will remain unclear, and strategies for facilitating implementation will remain elusive. This paper also contributes to recent calls for multilevel implementation interventions. By combining organization theories with theories that address factors at the level of individual or inner setting, we can develop high-leverage, multilevel implementation strategies rooted in well-established theories with extensive empirical support (44).

## Data Availability Statement

The datasets presented in this study can be found in online repositories. The names of the repository/repositories and accession number(s) can be found at cpcrn.org/resources.

## Author Contributions

JL, MW, MK, ML, MO'L, LK, and SB wrote case studies and theory overviews. JL wrote the introduction and discussion and combined all sections. MF contributed to the introduction and conclusion and provided feedback on the overall manuscript. All authors read and approved the final manuscript.

## Funding

This publication was funded by the Centers for Disease Control and Prevention of the U.S. Department of Health and Human Services (HHS) as part of a financial assistance award [cooperative agreement numbers: U48 DP006400, U48 DP006398, U48 DP006377, U48 DP006396] with 100 percent funded by CDC/HHS. The contents are those of the author(s) and do not necessarily represent the official views of, nor an endorsement, by CDC/HHS, or the U.S. Government.

## Conflict of Interest

The authors declare that the research was conducted in the absence of any commercial or financial relationships that could be construed as a potential conflict of interest.

## Publisher's Note

All claims expressed in this article are solely those of the authors and do not necessarily represent those of their affiliated organizations, or those of the publisher, the editors and the reviewers. Any product that may be evaluated in this article, or claim that may be made by its manufacturer, is not guaranteed or endorsed by the publisher.

## Abbreviations

5 A's: Ask, Advice, Assess Assist and Arrange
CHW: Community Health Worker
CPCRN: Cancer Prevention and Control Research Network
DHHS: Department of Health and Human Services
DSME: Diabetes Self Management Education
EBI: Evidence-Based Intervention
ERIC: Expert Recommendations for Implementing Change
FQHCs: Federally Qualified Health Centers
OTIS: Organization Theory for Implementation Science
RDT: Resource Dependency Theory
TCE: Transaction Cost Economics.
